# IPLaminator: an ImageJ plugin for automated binning and quantification of retinal lamination

**DOI:** 10.1186/s12859-016-0876-1

**Published:** 2016-01-16

**Authors:** Shuai Li, Michael Woodfin, Seth S. Long, Peter G. Fuerst

**Affiliations:** Department of Biological Sciences, University of Idaho, 145 Life Science South, Moscow, ID 83844 USA; Department of Computer Sciences, Lewis-Clark State College, Lewiston, ID 83501 USA

**Keywords:** Axon, Dendrite, Neuron, Brain, Band, Mosaic, Dscam, Bax

## Abstract

**Background:**

Information in the brain is often segregated into spatially organized layers that reflect the function of the embedded circuits. This is perhaps best exemplified in the layering, or lamination, of the retinal inner plexiform layer (IPL). The neurites of the retinal ganglion, amacrine and bipolar cell subtypes that form synapses in the IPL are precisely organized in highly refined strata within the IPL. Studies focused on developmental organization and cell morphology often use this layered stratification to characterize cells and identify the function of genes in development of the retina. A current limitation to such analysis is the lack of standardized tools to quantitatively analyze this complex structure. Most previous work on neuron stratification in the IPL is qualitative and descriptive.

**Results:**

In this study we report the development of an intuitive platform to rapidly and reproducibly assay IPL lamination. The novel ImageJ based software plugin we developed: IPLaminator, rapidly analyzes neurite stratification patterns in the retina and other neural tissues. A range of user options allows researchers to bin IPL stratification based on fixed points, such as the neurites of cholinergic amacrine cells, or to define a number of bins into which the IPL will be divided. Options to analyze tissues such as cortex were also added. Statistical analysis of the output then allows a quantitative value to be assigned to differences in laminar patterning observed in different models, genotypes or across developmental time.

**Conclusion:**

IPLaminator is an easy to use software application that will greatly speed and standardize quantification of neuron organization.

**Electronic supplementary material:**

The online version of this article (doi:10.1186/s12859-016-0876-1) contains supplementary material, which is available to authorized users.

## Background

Stratification of neural processes is a critical aspect of development that can promote specific patterns of connectivity and function. Many neuron cell types are also identified in part by the pattern in which their axons and dendrites stratify. The retina is part of the central nervous system where stratification, in the retina termed lamination, is perhaps most pronounced. Lamination of axons and dendrites occurs in the two neuropil layers of the retina, the relatively simply outer plexiform layer (OPL) and the more complex inner plexiform layer (IPL) [1]. The outer plexiform layer contains synapses between photoreceptors and cells of the inner retina, while the inner plexiform layer contains the synapses of inner retinal neurons and retinal ganglion cells, the output cells of the retina. The IPL is functionally and anatomically subdivided into ON and OFF halves, which generally contain synapses responsive to light (ON) or active in the absence of light (OFF).

In addition to its functional implications, the stratification pattern in the IPL is often used to identify and describe the population of the bipolar [2–4], amacrine [5–7] and retina ganglion cells [8, 9]. Lamination of the retina is also a commonly used parameter when evaluating the function of genes during development of the retina [10]. Current limitations to analysis of IPL lamination is the lack of a standard approach to quantifying laminar depth and the reality that mutations may result in changes to cell population densities that non-specifically alter the depth at which different populations of retinal neurons stratify. Automated approaches have been adopted to analyze optical coherence tomography images of the retina, but a standard approach to analyze lamination of retinal sections has not been developed [11]. In this manuscript we describe an application developed as an Image-J based plug-in that is directly aimed at solving this issue. This application significantly reduces the work-load involved in quantifying retinal lamination and automates demarcation of laminar depth based on biological features, removing biases introduced by different genetic backgrounds and human error. Additional features allow the user control of division (binning) of the IPL or other neural tissues based on user preferences.

## Implementation

### Software installation and operation

A detailed guide to the use of IPLaminator can be found in the additional files in the supporting information section (Additional files 1, 2, 3, 4, 5, 6 and 7: Figures S1-S7 and supporting information text (Additional file 8). Two sample images are included; S6 and S7). These files will walk the user through use of this software.

For quick reference the flow chart in Supplemental Figure 3 (Additional file 3: Figure S3) can be used: 1) The program prompts the user for a region of interest (ROI) containing the IPL and makes the rectangular selection tool the currently selected tool. 2) If “Add additional analysis region outside the IPL” is checked in the section menu the program prompts the user to select a point to the right of the already selected IPL ROI. This point represents the end of the additional analysis region (the region begins at the right edge of the IPL ROI). 3) If “Reduce background noise” is checked in the settings menu the program will prompt the user to select a background point on the image. The gray intensity is averaged at the selected point in a 3 by 3 region. This value is then saved and subtracted from the average intensity at each layer before the results are displayed. 4) If “Use percentile values to calculate layer boundaries” is selected in the settings menu, the positions of layer boundaries are calculated based on hard coded values. These values are represented as a percentile distance across the user selected ROI based on our measurement of SAC bands. The percentiles were determined experimentally and represent the average location of layer boundaries found in the IPL of wild type mice. 5) Depending on the stain type selected in the settings menu (ChAT or Calbindin/Calretinin) the program determines the location of two neurite stripes and a minimum between them or the location of three neurite stripes respectively. The process is illustrated in the following algorithm (Additional file 4: Figure S4). 6) If the layer boundaries are not being calculated using percentile values they are calculated using the boundaries of the IPL ROI and the locations of three biological markers described above. The layer boundaries (one through twelve) are calculated as described in the methods section. 7) After the layer boundaries are established the average intensity is calculated for each layer in each image selected for analysis. 8) At this point in the program all analysis is finished and results are generated. The following fields are automatically saved to a text file in the default output directory previously chosen by the user: • Layer Number – The given number of each layer, ascending from layer adjacent to RGC to layer adjacent to INL then additional area if selected. • Layer Depth – The location of each layer, the distance in pixel from the side of the IPL that borders the RGC. • Layer width – The width of a particular layer. • Intensity – Average gray scale intensity for each layer in each analyzed image. • Normalized Intensity – Normalized intensity is the average intensity for each layer/image minus 99 % of the lowest non-zero layer intensity value on that image. • Intensity minus background – The average intensity for a particular layer/image minus the average intensity in the 3x3 region around the user selected background point. Only output if reduce background noise is selected. • Intensity % - Intensity % is the intensity at a given layer divided by the intensity at all layers. 9) If “display results histogram” is selected in the settings menu histograms will be displayed to illustrate the results. One histogram is displayed for each individual image and a single histogram is displayed with the combined results from all images. These histograms are created with the ImageJ ProfilePlot class.

### IPL binning formula

The IPL is binned based on cholinergic amacrine cell neurite stratification (Additional file 5: Figure S5). Specifically, the two boundaries of the IPL are selected by the user, at the border of the IPL and the RGL and INL. 3 boundaries within IPL are generated based on the grayscale intensity profile of the cholinergic neurites, including 2 peak intensity locations and a lowest intensity location between these two peaks. These five locations are used to generate 10 sublayers.

The distance between the inner boundary of IPL (adjacent to RGL) and the location of the ON peak intensity is divided into 7 layers. Two of these layers starting from the RGL are then merged into a sublamina, which gives 3 sublamina in the bottom of the ON layer (layer 1–3). The 7th layer is adjacent to the peak intensity of the ON cholinergic band facing RGL and this layer is added to 1/4 the distance towards the location of lowest intensity between the peaks from layer 4 and covers most ON cholinergic band. The next 1/2 of the distance between the ON cholinergic band and the middle point between the two cholinergic neurite bands is layer 5. The last 1/4 of the distance is added to 1/4 of the distance towards the OFF cholinergic peak intensity and defines layer 6. Next 1/2 of the distance between the least intensity between the cholinergic neurite bands and the OFF cholinergic peak intensity is defined as layer 7. The last 1/4 of the distance adjacent to OFF cholinergic peak is added to 1/5 of the distance towards the outer boundary of the IPL, and forms layer 8, covering most of the OFF cholinergic band. The remaining 4/5 of the distance towards the IPL boundary is divided into 2 layers of equal thickness; layer 9 and 10. Each of these divisions represents close to 10 % of the IPL, as shown in the results section.

## Material and methods

### Animal care and handling

Ad libitum fed mice were maintained on a mixed C57 BL/6 J and C3H/HeJ background under a 12 h light:dark regimen. Wild type, *Dscam*^*LOF*^ and *Bax*^*−/−*^ mutant mice [12] were used in this study. All animal procedures performed on mice in this study were approved by the University of Idaho Animal Care and Use Committee. Genotyping was performed as previously described according to instructions from The Jackson Laboratory.

### Immunocytochemistry, immunohistochemistry and antibodies

Mice were perfused with PBS. Whole eyes were marked by making a small burn on the dorsal side of the corneal and then fixed in 4 % PFA for 2 h at room temperature and washed overnight. Retinas were then cryo preserved, frozen in optimal cutting technology (OCT) media (Tekura Inc) at −20°. Tissue was stained as previously described [13].

Antibodies: goat anti-ChAT (Millipore; AB144P; 1:400), rabbit anti-calbindin (Swant; CB38a; 1:1000), rabbit anti-bNOS (Sigma-Aldrich; NZ280; 1:15,000), rabbit anti-TH (Millipore Bioscience Research Reagents; 1:500), mouse anti Syt2 (ZFIN; ZDB-ATB-081002-25; 1:500). DAPI reagent was mixed into the second wash after incubation with secondary antibodies at a dilution of 1:50,000 of a 1 mg/ml stock. Secondary antibodies were acquired from Jackson ImmunoResearch and used at a concentration of 1:1000.

### Microscopy

An Olympus DSU confocal microscope was used to capture all fluorescent images. 20 X and 40 X objectives were used in this study with numerical aperture of 0.5 and 1.2 respectively. The final image resolutions were 0.4 and 0.2 μm per pixel. A Nikon epifluorescent microscope was used to capture images of H&E sections. To avoid immunoflourescent background, exposure rate was set on auto to minimize the noise from different channels. Images used in the figures were taken at exposure rate of 100 ms. Any modification to images, for example, to brightness, was performed across the entire image in accordance with the journal’s standards. Similar results were obtained using images collected from a variety of imaging platforms on our campus; all fluorescent microscope camera combinations captured sufficient images for analysis.

## Results

### Convention of IPL strata division

**Fig. 1:**
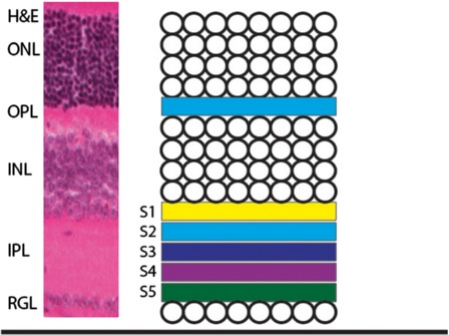
Organization of the retina. The retina is divided into three nuclear layers and two synaptic layers. The nuclear layers are the ONL (outer nuclear layer), INL (inner nuclear layer) and RGL (Retinal Ganglion Layer). The synaptic layers are the OPL (outer plexiform layer) and IPL (inner plexiform layer). The IPL is conventionally divided into five strata, S1 to S5

Ramon y Cajal pioneered the convention of subdividing the retina’s IPL [15]. He divided the IPL into five layers based on the transverse processes of Müller glia, termed strata S1-S5 (Fig. 1). An adaptation of this convention is widely used, with the IPL divided into 5 even layers based on IPL thickness, rather than the location of Müller glia transverse processes. This method of subdividing the IPL is widely used to quantify the lamination of retinal neurites, with some researchers later adopting 10 strata [16]. For example, bipolar cells in the mammalian retina have been categorized into 12 different types based in part on the depth of their axon projections in the IPL and similar classification is widely used to classify retinal ganglion and amacrine cells [5, 7, 17]. Division of the IPL into distinct layers is also a useful convention to follow because retinal neuron types project axons and dendrites to molecularly defined layers of the IPL and this is required for their function [18].

**Fig. 2:**
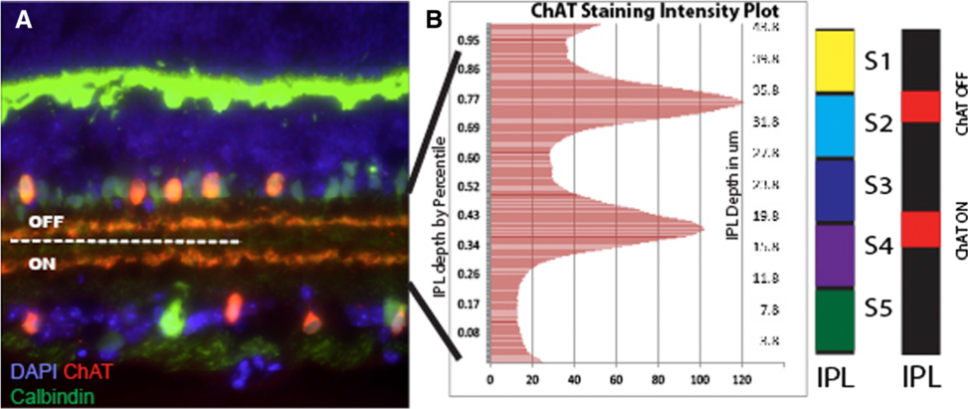
Synaptic organization of the inner plexiform layer. **a** The IPL is functionally divided into ON and OFF halves. The starburst amacrine cell (SAC) neurite bands are prominent features in either half of the IPL. **b** Plotting staining of SAC bands S1-5 reveals two intensity peaks (*left*). Neither of the two peaks fall into stratum as defined by the conventional method of divided the IPL (*right*). Scale bar (in A) = 100 μm

Functionally the IPL is divided into ON and OFF halves [19, 20] (Fig. 2a). A prominent landmark in many species’ retina is a paired set of cholinergic starburst amacrine cell (SAC) neurites [6, 21, 22] (Fig. 2a), referred to as the bands of these cells. When the intensity of ChAT staining in the IPL is plotted out these two bands emerge as paired histogram peaks (Fig. 2b). ChAT expression in the mouse retina is first observed at embryonic day 18 and two distinct ChAT-positive bands emerge at early postnatal time points [23]. SACs project into two clearly separated strata where their synaptic connections are responsible for ON and OFF stimuli mediated direction selectivity [24–26]. When plotted against the five conventional IPL bands, the ON SAC band mapped between S3 and S4, with a location approximate 40 percentile in depth from RGL, and span about 10 % of the total IPL thickness (*n* = 19, SD = 1.2 %) (Fig. 2b). The peak intensity of the OFF SAC band was located approximately 77 percentile in depth from the RGL and spanned close to 10 % of the total IPL thickness (*n* = 19, SD = 1.4 %) (Fig. 2b). The point in between these bands, marked by antibodies to calbindin or calretinin, faithfully demarcates the ON and OFF halves of the retina, close to 60 % of the IPL distance from the RGL (Fig. 2a).

Given the real physiological landmarks identified by cholinergic amacrine cell banding, these observations suggest that demarcating the IPL into five even layers roughly based on projection of Müller glia may not best represent the biology of the retina. Using cholinergic bands to demarcate and subdivide the ON and OFF halves of the IPL offers an attractive solution to this problem by functionally dividing the retina into a similar series of domains as envisioned by Cajal, with the added benefit of an easily reproducible set of landmarks. Further division allows the identification and distinction of spatially separated but distinct neurites, which would otherwise be classified as projecting to the same stratum of the IPL.

### Automated strata delimitation of the IPL

**Fig. 3:**
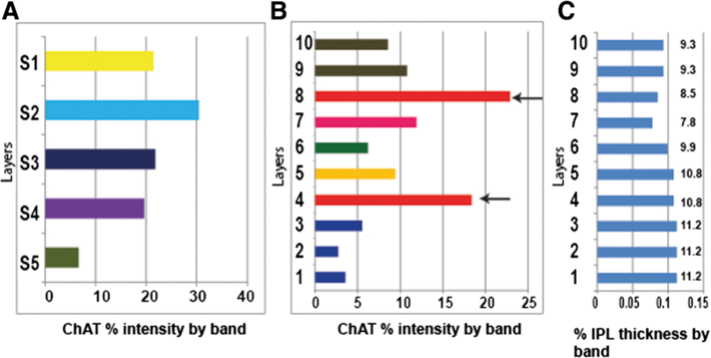
Distribution of ChAT staining according to custom and novel binning methods. **a** ChAT staining peaks are difficult to discern when the retina is dividing into five conventional strata. **b** Binning of the IPL into ten bands based on location of peak SAC band intensities. SAC bands are clearly visible. **c** Each of the ten strata in this classification scheme represents about 10 % of the IPL

To develop a reproducible automated system on which to develop a binning tool we started with the nuclear layers and the SAC neurite bands to begin subdividing the IPL. Conventional division of the IPL into five strata results in the division of cholinergic staining divided among the five strata (Fig. 3a). The software we developed uses the margins of the IPL and INL or RGL, the peak intensity of each cholinergic band and the local minimal intensity between the two cholinergic bands to divide the IPL into ten bins (Fig. 3b; see Methods for a detailed description of how each band is calculated). Each of the ten bands represents approximately 10 % of the IPL in the mouse retina (Fig. 3c). The traditional method of IPL binning was built into the program to allow users to bin the IPL into a user-defined number of bands, useful for example in cases where cholinergic banding is disrupted. We also added an option to bin the IPL based on our measurements of cholinergic IPL stratification. Another alternative method to manually select band number and location was also added and will allow for binning cortex, zebrafish retina (which has three SAC bands), or to bin other regions of interest based on user needs.

### Analysis of abnormal neuron stratification

**Fig. 4:**
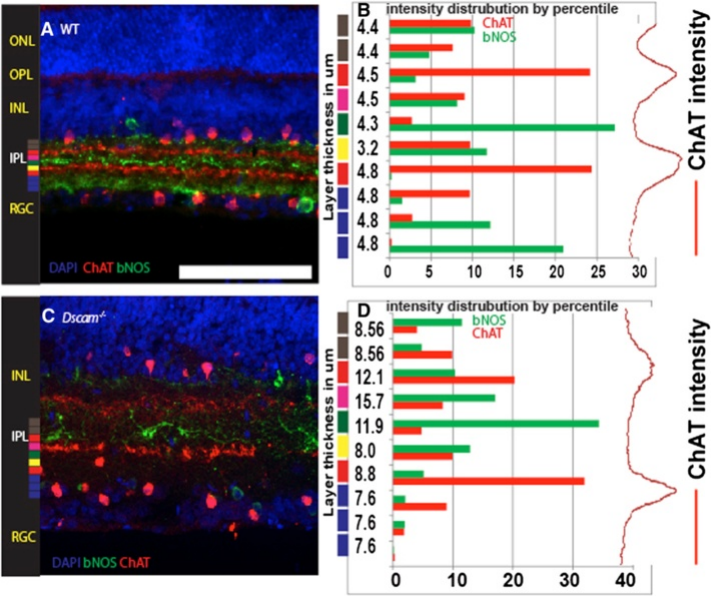
IPLaminator binning of wild type and mutant retinas. **a** Image of a wild type retina section stained with antibodies to ChAT and bNOS and the nuclear stain DAPI. **b** Division of the IPL into ten bands with ChAT and bNOS staining intensities plotted in each band. **c** Image of a *Dscam* mutant retina section stained with antibodies to ChAT and bNOS and the nuclear stain DAPI. **d** IPLaminator was able to accurately bin the IPL despite the presence of multiple local peaks within the ON or OFF band. Scale bar (in A) = 140 μm

The location of SAC bands is used by IPLaminator to bin the IPL into a defined number of layers (Fig. 4a and b). In some mutant strains; however, the SAC bands begin to disperse and we wanted to confirm our program could still automatically identify both bands. We have previously demonstrated defects in SAC banding in the *Dscam* mutant retina and used this genotype to test the ability of our software to demarcate the IPL (Fig. 4c). The algorithm we applied (for cholinergic bands by ChAT staining) would seek two peak intensities starting from the middle of the IPL. This allowed the software to successfully identify both ON and OFF SAC bands even when multiple local peaks were observed in either the ON or OFF band (Fig. 4d).

### Analysis of neuron projection outside of the IPL

**Fig. 5:**
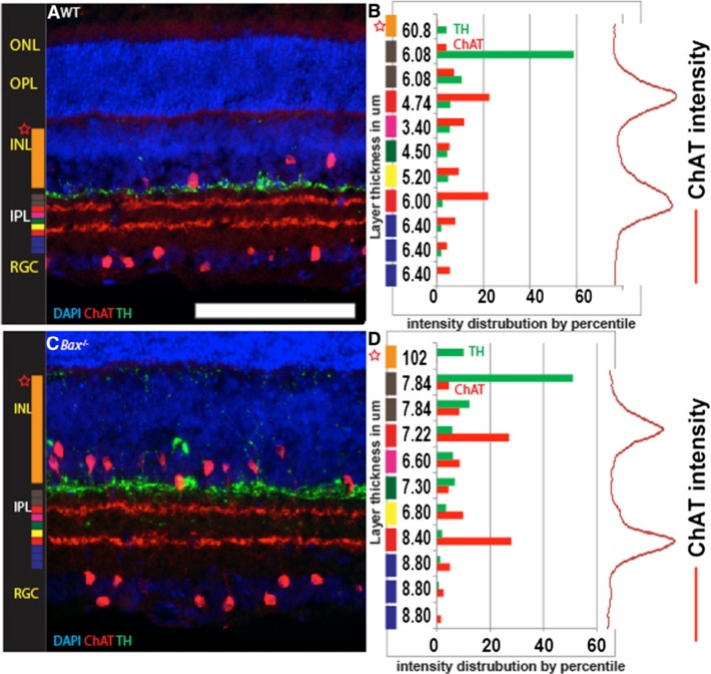
IPLaminator for calculation of misprojected neurites. **a** Section of wild type retina stained with antibodies to tyrosine hydroxylase (TH) and ChAT, and the nuclear dye DAPI. **b** IPLaminator readout of TH staining intensities in each of 10 stratum and in the INL as a percent of total staining. **c** Section of *Bax* mutant retina stained with antibodies to tyrosine hydroxylase (TH) and ChAT, and the nuclear dye DAPI. **d** IPLaminator readout of TH staining intensities in each of 10 stratum and in the INL as a percent of total staining. Scale bar (in A) = 140 μm

In some situations, both normal and pathological, neurons project processes outside of the IPL. For example, type 1 dopaminergic amacrine cells project a small number of axons to the outer plexiform layer (OPL) (Fig. 5a and b). In mutant genetic backgrounds, for example *Bax* null mice, TH positive amacrine cells send an increased number of axons to the OPL (Fig. 5c) [12]. In order to measure neurites projecting outside of the IPL, we added an option to calculate the amount of staining in a user-defined region spanning from the INL/IPL boundary to a set point. The program then measures the set area and calculates the percent intensity of the measured stain compared to total intensity (Fig. 5d). We can see a clear difference in the image based quantification data for TH. The INL displays 10 % of the IPL intensity in the mutant retina located within the INL compared to less than 2 % in the wild type image.

### Alternatives to ChAT staining

**Fig. 6:**
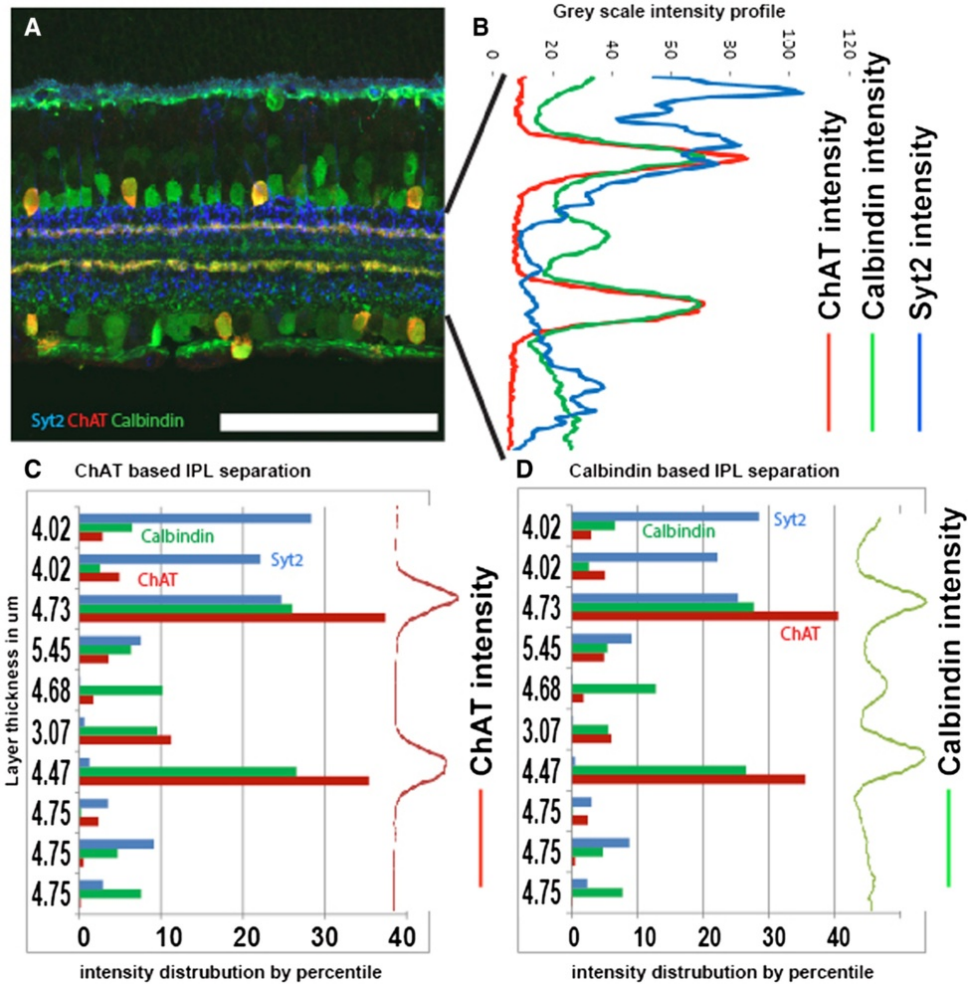
Calbindin as an alternative to ChAT for strata demarcation. **a** Section of wild type retina stained with antibodies to Syt2, ChAT and calbindin. **b** Gray scale intensity profiles for Syt2, ChAT and Calbindin. **c** Use of ChAT based layer separation on three channels. **d** Use Calbindin based layer separation on three channels. We see very marginal difference between the two mainly the middle retina layers. Scale bar (in A) =105.8 μm

Limitations in antibody compatibility can limit the combinations of cells that can be stained and we therefore tested if an alternative to ChAT could be identified. Calbindin and calretinin are calcium binding proteins that label a mixed population of retinal neurons, including the SACs and their neurite bands (Fig. 6a and b). We added an option to run IPLaminator using calbindin or calretinin staining as a guide that accounts for the band intermediate to the two SAC bands that is stained by both of these antigens. Using this modification IPLaminator is able to utilize calbindin as an alternative to ChAT and generated stable layer separation that is very similar to using ChAT (Fig. 6c and d).

## Discussion

IPLaminator is a simple tool with a wide range of uses for analysis of lamination in the retina and other regions of the central nervous system. The data output of IPLaminator is primarily in percent values and reflects the amount of fluorescent intensity in a given layer of neural tissue. This output can then be statistically compared across genotypes using a statistical test optimized for comparison of percents, such as the Mann–Whitney U-test, or converted, for example by arc-sin conversion, for other statistical tests.

### Biological limitations and considerations

Several biological considerations and limitations should be taken into account when assaying retinal lamination. The first of these is that the eye is a spherical structure and this analysis treats lamination across a flat plane. The angle at which the retina curves and thins from the central retina to the peripheral retina is small in adult mice but at earlier developmental stages and in models such as zebrafish larva the angle is greater and could result in the artifactual smearing of sharp lamination across multiple bands. A solution to this bias is to sample a smaller distance of IPL more frequently (to account for increased variability over a smaller distance).

Antibody staining quality is an obvious complication and can result in signal being spread over portions of the IPL that clearly do not have neurites projecting into them. IPLaminator measures intensity of fluorescence and not neurite projections per se, with the assumption that most staining will be concentrated in targeted neurites. Background subtraction across the image using the program’s background subtraction or before analysis can reduce the influence of background immunofluorescence but care must be taken to ensure inappropriate image manipulation does not occur at this stage, which could result in greater background subtraction from some subset of tissues. In practice we code genotypes and cut sections from different genotypes to be analyzed onto the same slide for staining. This helps to blind the analysis and minimize sample-sample preparation variability.

The presence of displaced cell bodies in the IPL can complicate analysis in several manners. The most distorting of these is if the soma is itself fluorescent. This would result in a large signal in the stratum in which the soma resided. Avoiding such areas or recognizing that the signal is coming from the cell soma is a necessary consideration. Two classes of cells that normally reside in the IPL include the soma of vasculature and microglia. Both of these cells have a tendency to nonspecifically fluoresce, especially when using antibodies generated in the species to be assayed.

The axon and dendrite stalk projecting to laminated neurite bands is also a consideration. These processes contribute to readout of signal and their differential staining in compared populations could result in mistaken interpretation of data. Using antibodies that limit this will increase resolution. For example antibodies to VAChT stain the SAC bands only, while ChAT stains the cell bodies and proximal and distal neurites of SACs [26]. In cases where cell bodies are not displaced into the IPL either ChAT or VAChT will yield similar results because the peak intensities are used to bin the IPL. In cases where somata are displaced into the IPL antibodies to VAChT will avoid picking up signal from the displaced cell bodies in the IPL.

### Technical limitations and considerations

Regarding background noise, both original intensity or with background subtraction, the software automatically detected minimal background intensity and intensity generated by user-selected background and all will be used to generate three clusters of results. The only differences between three outcomes is how much intensity has been removed from each channel globally because our software could not distinguish a pixel that is labeling neurons to a pixel that is a pure background noise. Users should carefully examine the outcomes and consistently use one of the three results to interpret original data.

## Conclusion

IPLaminator is designed to optimize IPL neurite stratification analysis. It minimizes human operational error and observational bias, generates reliable and accurate data based on individual images to best describe how neurons projecting their neurites. Use of IPLaminator is intuitive with minimal amount of training time required. Once the image is set up correctly, users only have to select an area of interest and the software will automatically optimize the layer separation based on intensity displayed throughout the area. IPLaminator represents a technical and scientific improvement on Cajal’s early studies of the retina that will help to continue the mapping of the nervous system he started 120 years ago.

## Availability and requirements

Project name: IPLaminatorProject homepage: http://isoptera.lcsc.edu/IPLaminator

Operating system: Windows, Mac, and Linux.

Programming language: R, Python and Java.

Other requirements: Image J or FIJI (Image J with auto plugin update version) is required for this program to run.

License: This program is free software: you can redistribute it and/or modify it under the terms of the GNU General Public License as published by the Free Software Foundation, version 3 of the License.

Any restrictions to use by non-acadamics: None

## Additional files

Additional file 1: Figure S1. IPLaminator interface. A, Angle tool was selected to determinate rotation and the degree was displayed in the Fiji interface under tool selection area. B, Rotation tool was used to rotate image so that the RGC layer is facing left and the retina is vertically aligned. C, User interface of IPLaminator, it simply asks user to define 3 elements. First, the image used to define nuclear layer boundaries. Second S2/S4 Plot Profile is an image of ChAT or equivalent staining that will be used to automatically define layers. Last, all image channels that need to be analyzed are selected. Additional file 2: Figure S2. A, Area selection. Once the image is set up, only one operation is required, that is to select the region of interest (ROI). B, Setting Interface for user to set up parameters and other system functions. Additional file 3: Figure S3. Flow chart. Additional file 4: Figure S4. Key algorithms used in layer separation. A, Code to determine how the signature peaks are located. B, How each of the ten stratum are generated (complementary to Additional file 5: Figure S5). C, Preset values used in “Use percentile value” function to bin IPL. Additional file 5: Figure S5. Binning of the IPL based on location of cholinergic amacrine cell neurites. Scale bar = 30 μm. Additional file 6: Figure S6. An image users can download and practice with. ChAT is stained in the red channel and TH is stained in the green channel. Additional file 7: Figure S7. An image users can download and practice with. Calbindin is stained in the red channel and TH is stained in the green channel. Additional file 8:
**Supporting Information.**
