# Supplementary figures and images for: IPLaminator: an ImageJ plugin for automated binning and quantification of retinal lamination
Source: BMC Bioinformatics. 2016 Jan 16;17:36. doi: 10.1186/s12859-016-0876-1 (PMC4715356; doi:10.1186/s12859-016-0876-1)

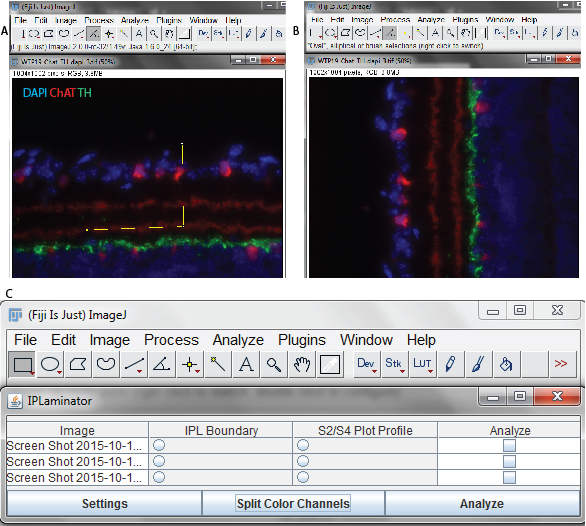

Supplement: Additional file 1: Figure S1. — IPLaminator interface. A, Angle tool was selected to determinate rotation and the degree was displayed in the Fiji interface under tool selection area. B, Rotation tool was used to rotate image so that the RGC layer is facing left and the retina is vertically aligned. C, User interface of IPLaminator, it simply asks user to define 3 elements. First, the image used to define nuclear layer boundaries. Second S2/S4 Plot Profile is an image of ChAT or equivalent staining that will be used to automatically define layers. Last, all image channels that need to be analyzed are selected. [file 12859_2016_876_MOESM1_ESM.jpg]

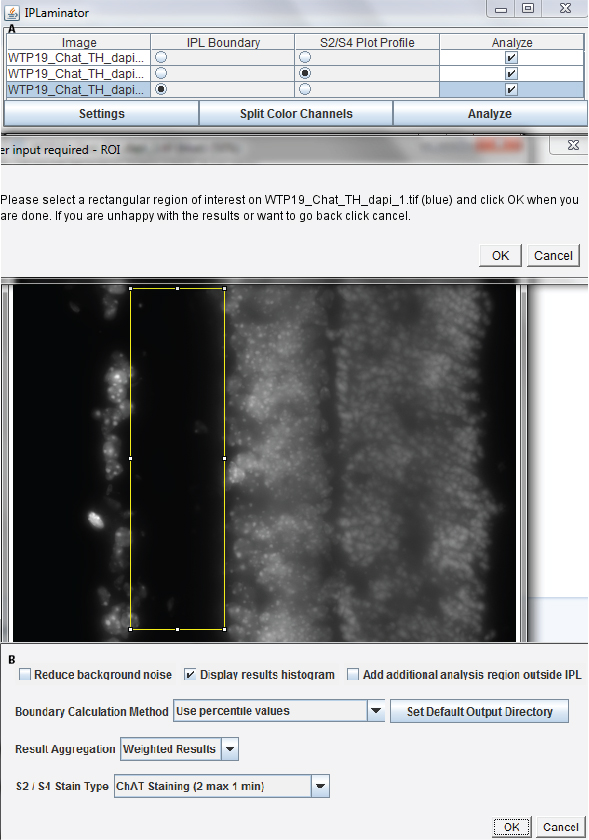

Supplement: Additional file 2: Figure S2. — A, Area selection. Once the image is set up, only one operation is required, that is to select the region of interest (ROI). B, Setting Interface for user to set up parameters and other system functions. [file 12859_2016_876_MOESM2_ESM.jpg]

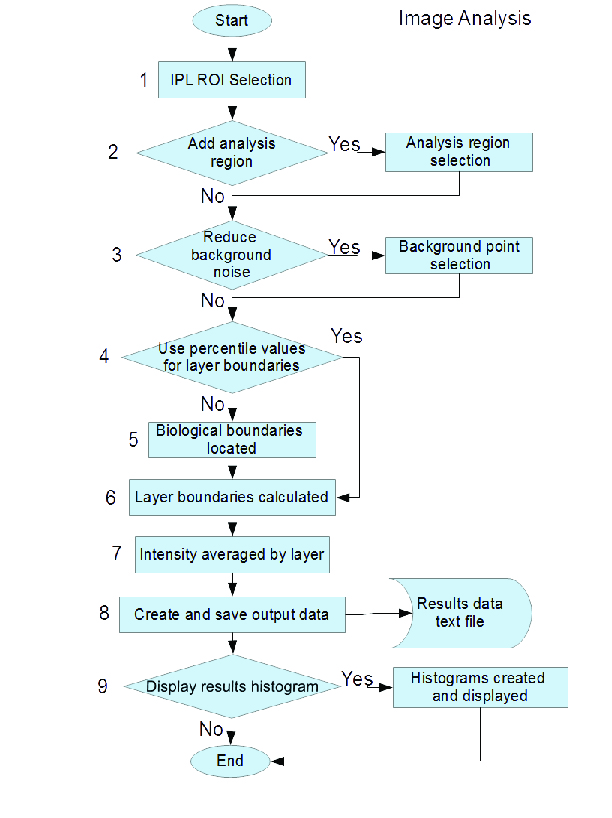

Supplement: Additional file 3: Figure S3. — Flow chart. [file 12859_2016_876_MOESM3_ESM.jpg]

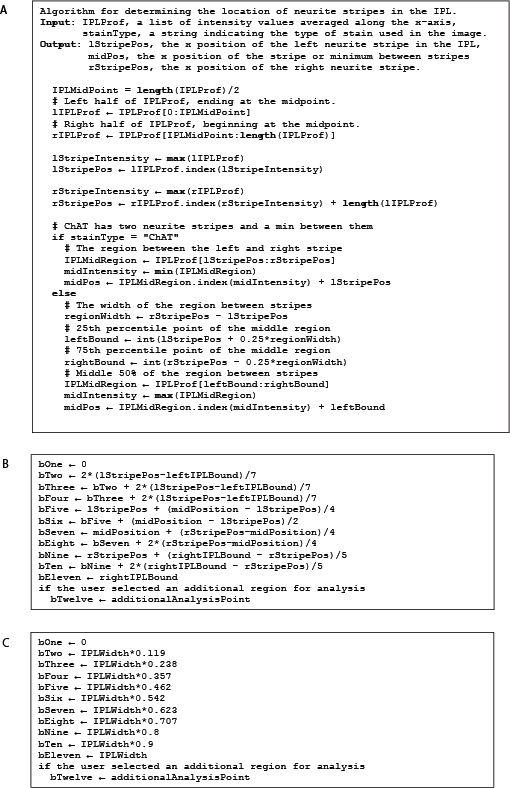

Supplement: Additional file 4: Figure S4. — Key algorithms used in layer separation. A, Code to determine how the signature peaks are located. B, How each of the ten stratum are generated (complementary to Additional file 5: Figure S5). C, Preset values used in “Use percentile value” function to bin IPL. [file 12859_2016_876_MOESM4_ESM.jpg]

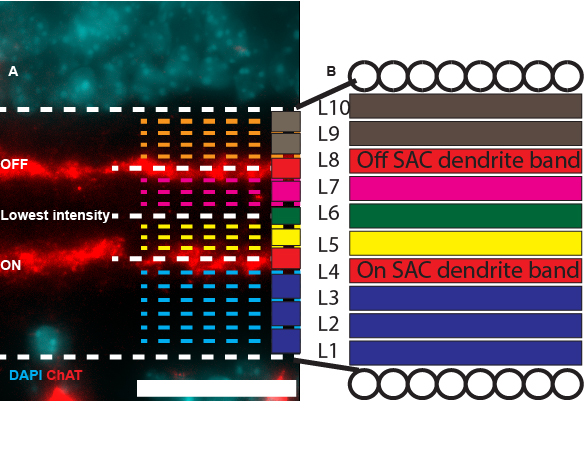

Supplement: Additional file 5: Figure S5. — Binning of the IPL based on location of cholinergic amacrine cell neurites. Scale bar = 30 μm. [file 12859_2016_876_MOESM5_ESM.jpg]

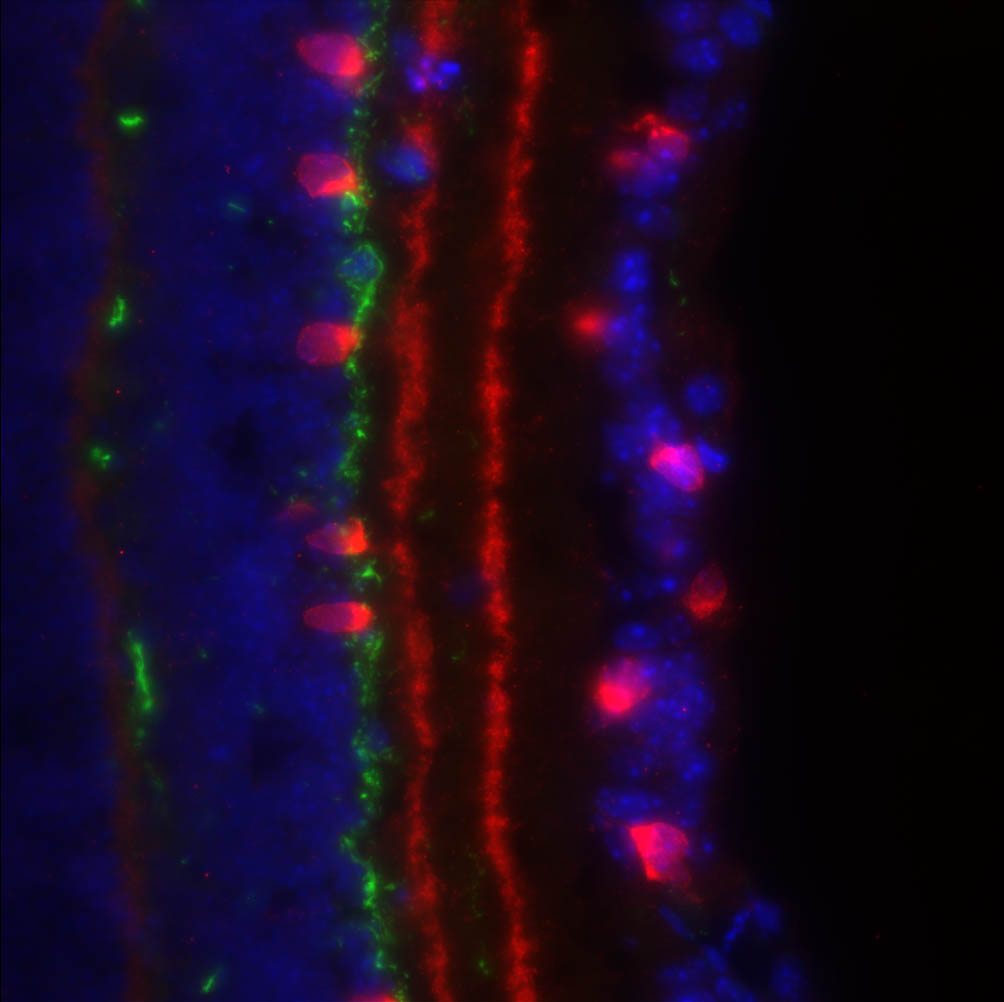

Supplement: Additional file 6: Figure S6. — An image users can download and practice with. ChAT is stained in the red channel and TH is stained in the green channel. [file 12859_2016_876_MOESM6_ESM.jpg]

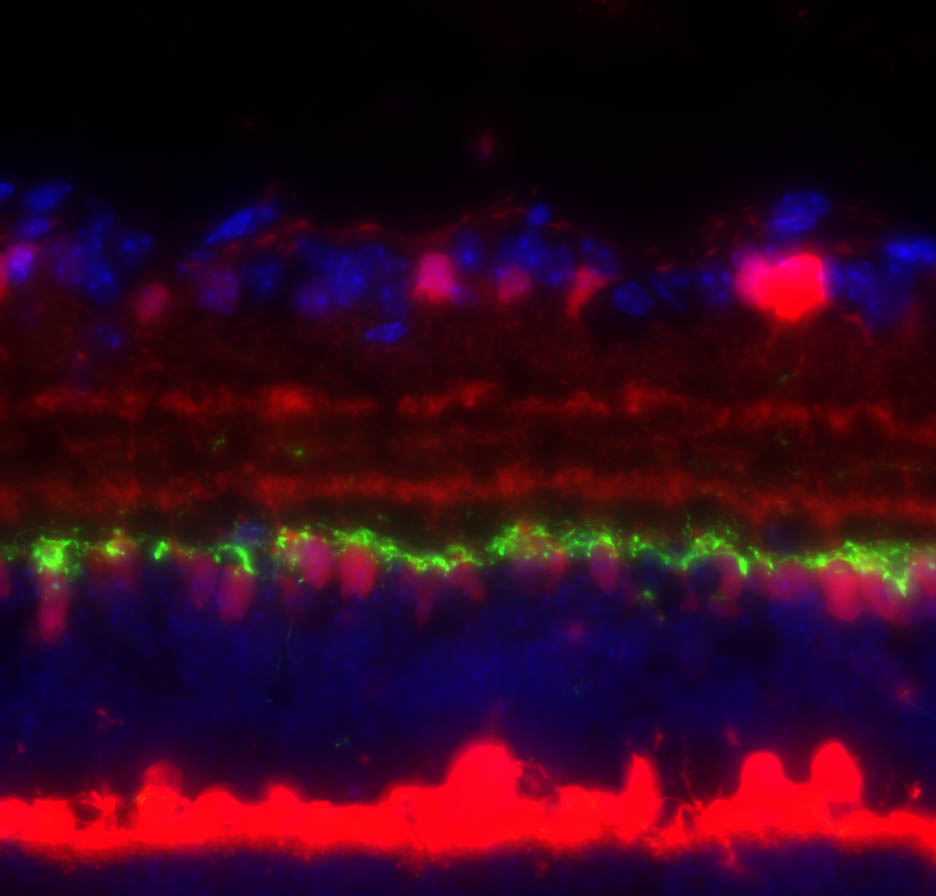

Supplement: Additional file 7: Figure S7. — An image users can download and practice with. Calbindin is stained in the red channel and TH is stained in the green channel. [file 12859_2016_876_MOESM7_ESM.jpg]
