# Supplementary material for: IPLaminator: an ImageJ plugin for automated binning and quantification of retinal lamination
Source: BMC Bioinformatics. 2016 Jan 16;17:36. doi: 10.1186/s12859-016-0876-1 (PMC4715356; doi:10.1186/s12859-016-0876-1)
Supplement: Additional file 8: — Supporting Information. [file 12859_2016_876_MOESM8_ESM.docx]

**Supporting Information**

**Installation:** The freeware package ImageJ or FIJI is required before the installation of IPLaminator. The IPLaminator plugin can be downloaded from the following website <http://isoptera.lcsc.edu/IPLaminator>. Once the python file is downloaded (you will need to right click and use the save as function), place the file into the ImageJ (or FIJI) plugins folder and restart the program. Then under the Plugins choose install plugin and select the downloaded file for ImageJ/FIJI to install. IPLaminator will appear under the plugins section after installation. Restarting the software after installation may be necessary on some platforms, for example on Mac computers.General Instruction: An RGB image of retina or other tissue to be analyzed should be opened in ImageJ/FIJI. The first step is to orient the tissue for analysis. The retinal ganglion cell layer (RGL) must face left and should be vertically orientated (Supplemental Figure 1 B). Use the angle tool in the main tool bar selection tab to determinate the angle of rotation necessary to orient the image for analysis if not already aligned (Supplemental Figure 1 A), this angle can be used to orient the retina using the rotation tool in ImageJ under image, transform. The region of interest (ROI), the IPL, should run vertically. Most imaging software translates total intensity of light within a given wavelength into red/green/blue output. In order to analyze the intensity of each color channel the RGB image information should be split into separate gray scale files. The relative shade of gray- from black to white is measured and plotted out in the histogram files. Use the Split Color Channel function in the software to separate the RGB image into 8-bit gray scale images. The intensity of the gray scale images from each channel is then analyzed through our program. This function can be found under Image-Color-Split Channels in Image J.

To operate the software, open IPLaminator under plugins in ImageJ. It will bring up the user interface (Supplemental Figure 1 C). Only 3 settings are required to complete the analysis. First choose the color channel that will be used to define the IPL boundary. This can be done with a nuclear stain where the IPL layer is the nucleus free zone between the inner nuclear layer (INL) and RGL (DAPI imaged in blue in the sample image). The second step is to define a color channel to be used to identify ON and OFF starburst amacrine cell neurites in the S2/S4 plot profile channel (ChAT stained red in the sample image). If these neurites are not present in the image other options are available and will be discussed in the next section. In the third step the user should select all images to be analyzed. Options under the settings tab will be discussed in the following section. Press the analyze button. If no directory has been set up, the program will have a mandatory warning and the user will be asked to set up a directory for the output files before further operation.

After selecting analyze a box prompting the user to select the ROI will appear. Select the IPL ROI using the rectangular selection tool using the DAPI nuclear staining in the sample as a guide (Supplemental Figure 2 A). Select an area that represents the retina lamination pattern to be analyzed and includes the entire depth of the IPL from the RGL to INL. The ROI not only chooses the region of interest but also sets the boundary between the IPL and nuclear layers flanking the IPL. IPLaminator uses parameters extracted from the IPL ROI and the peak intensity of the cholinergic neurites (or user defined methods which will be discussed) to bin the IPL into reproducible fractions and to calculate the gray scale intensity for that color channel. The area of analysis is identical in all color channels. At least one channel has to be selected as directed in step three, (above; images to be analyzed) in order to generate an output.

Data in the result file is listed by type for each column, with IPL layers stacked in rows. Data types include layer number, depth, original intensity profile by layer, original intensity percentile, automatically normalized intensity profile by layer, automatically normalized percentile, normalized intensity profile by user defined background subtraction (if user selected this option), normalized user subtracted percentile (if user selected this option), and layer width.

**Additional Features:** Other options in settings are available to address specific needs (Supplemental Figure 2 B).

**Reduce Background Noise:** If this function is enabled, an additional window appears after the select ROI task. This box will prompt the user to identify an appropriate background area using a point tool. Select an area that will be used for background subtraction, for example an area of the image without specific staining or fluorescence, by clicking in the area that best represents the background. The selected point will average the gray values of with adjacent pixels (3 by 3) and this value will be subtracted to reduce the signal to noise ratio by subtracting from the global IPL values. To prevent operational failure that could occur if the selected noise is above the threshold of the signal, the software calculates the lowest gray value from the ROI and uses it as a base line subtraction value.

Thus, three sets of result clusters will be generated for each program run, the first is the original intensity profile without alternation, the second is based on the base line value named “Normalized”, in which the lowest background level in the ROI is automatically used as a background subtraction level and a third cluster, which is based on the user selected background noise, named “minus_background”.

**Display Results Histogram**: The second settings feature is “Display results histogram”. This feature does not require any operation but will simply display a set of color histograms to illustrate the results to the user. This feature enables the user to visualize the output and data quality. One histogram is displayed for each individual channel and a compiled histogram is displayed with the results from all images with matching colors. These histograms are created with the ImageJ ProfilePlot class and must be saved if the user wishes to retain this information.

**Add Additional Analysis Region Outside IPL**: The third settings feature is “Add additional analysis region outside IPL”. This feature allows the user to select a new boundary with a point tool, for example to calculate the number of neurites within the inner nuclear layer, as described in the results section. This new boundary will define a new area as an additional layer between IPL outer boundary and the manually selected boundary. Gray scale intensity values are averaged through this entire area.

**Boundary Calculation Method**: Besides using the cholinergic amacrine cell neurites as biological markers to accurately represent each image, we programmed three alternative solutions when biological markers are unavailable or not applicable. These three functions are preset percentile based layer separation, user defined strata number separation and user defined strata locations separation.

**Use biological markers**: The default method IPLaminator uses to bin the IPL is based on the peak intensity of paired starburst amacrine cell neurites in the ON and OFF halves of the retina. This method determines the maximum intensity of the ON or OFF neurites and divides the IPL based on these locations and the inner and outer margins of the ROI.

**User Percentile Values**: An option to use a preset percentile is available based on measurements we have performed using the ON and OFF starburst amacrine cell neurites. The preset percentiles were calculated based on cholinergic banding in 19 WT retinas at an age of 20 days; use this parameter when the experiment is suitable.

**N Equal Boundaries**: If the user wishes to bin the IPL in the traditional convention of 5 even strata, or some other number of strata, this option allows the user to define the number of strata into which the ROI will be split. The user defined band number will simply divide the IPL into the given number of layers and calculate intensity accordingly.

**User Defined Boundaries**: Finally we give an option for the user to manually determine how the ROI will be binned. This will allow the user to manually set boundaries within the IPL, or other tissue, from their point of view, and intensity will be calculated accordingly. This could be useful for binning the IPL of zebrafish, which has more than two layers of cholinergic neurite, or to bin other tissues, such as cortex.

**Set Default Output Directory**: This function allows the user to set where the results are saved. The result files will be named after each image with the date that it was taken.

**Result Aggregation:** This function is to assist the quantification of lamination in different tissue shapes, for example curved tissue. One of the three following methods can be selected, Default Results, Average Results, and Weighted Results.

**Default results**: This is the default option and corresponds to the standard method of analysis that has been previously described. This is ideal to be used on tissues with straight lamination patterns and minimal curvature.

**Average Results**: This option enable user to analyze more curved tissue samples by selecting smaller ROIs over a large area then give equal weight to each region the user selects for analysis regardless of the region’s size. The user should perform an analysis as normal but instead of automatically saving the results after first analysis, the program asks the user if they would like to analyze another region or have the results collected up to this point output. If the user chooses to analyze another region, then they are first allowed to rotate the image, or whatever else is necessary to prepare the next region on the image to align with the analysis standards and then perform the analysis as normal. This process repeats until the user decides to save the results. This type of analysis could be useful for imaging curved sections of retina or other tissue where the user wishes to sample multiple points.

**Weighted Results**: Similar to Average Results but the results weigh the size of the user selected regions in the final result by using the following formula: ∑ (height x intensity)/∑ height.

**S2/S4 Stain type**: The starburst amacrine cell neurites used as landmarks in IPL laminator can be stained with other antibodies in addition to ChAT, including VAChT, calbindin or calretinin. Retinas stained with VAChT will generate a profile using the algorithm for ChAT. To use calbindin or calretinin to bin the IPL we modified the formula used because of the presence of an intermediate neurite band between the two ON and OFF cholinergic neurites. The user can therefore use alternative markers such as calbindin and calretinin if this feature is selected.

The overall program flow chart demonstrates how each function is operated upon the selection of ROI (Supplemental Figure 3). The software reads the image intensity profile and locates 3 signature locations (Supplemental Figure 4 A), then utilizes boundary locations and signature locations to separate IPL into 10 uneven layers (Supplemental Figure 4 B), each roughly ten percent of the IPL. When image based layer separation is unavailable, we provided alternative functions including percentile based layer separation (Supplemental Figure 4 C), user defined layer separation and user defined locations separation to allow users customize the parameter of measurement.

When the parameter is established, the gray scale intensity profile is measured for each color and averaged for each layer, and then a series of results are calculated and displayed both in txt format and histogram if this option is enabled.
